# Membrane Processes for the Nuclear Fusion Fuel Cycle

**DOI:** 10.3390/membranes8040096

**Published:** 2018-10-12

**Authors:** Silvano Tosti, Alfonso Pozio

**Affiliations:** 1 Department FSN, ENEA, C.R. Frascati, Via E. Fermi 45, Frascati, 00044 Rome, Italy; 2 Department DTE, ENEA, C.R. Casaccia, Via Anguillarese 301, S. Maria di Galeria, 00123 Rome, Italy; alfonso.pozio@enea.it

**Keywords:** Pd-membranes, hydrogen isotopes, fusion fuel cycle

## Abstract

This paper reviews the membrane processes for the nuclear fusion fuel cycle—namely, the treatment of the plasma exhaust gases and the extraction of tritium from the breeding blankets. With respect to the traditional processes, the application of membrane reactors to the fusion fuel cycle reduces the tritium inventory and processing time, thus increasing the safety and availability of the system. As an example, self-supported Pd-alloy membrane tubes have been studied for the separation of hydrogen and its isotopes from both gas- and liquid-tritiated streams through water-gas shift and isotopic swamping reactions. Furthermore, this paper describes an innovative membrane system (Membrane Gas–Liquid Contactor) for the extraction of hydrogen isotopes from liquid LiPb blankets. Porous membranes are exposed to the liquid metal that penetrates the pores without passing through them, then realizing a gas–liquid interface through which the mass transfer of hydrogen isotopes takes place. Compared to the conventional hydrogen isotope extraction processes from LiPb that use the “permeator against vacuum” concept, the proposed process significantly reduces mass-transfer resistance by improving the efficiency of the tritium recovery system.

## 1. Introduction

In long-term scenarios, nuclear fusion is thought to provide carbon-free energy via the exploitation of magnetic confinement devices (tokamaks), where the nuclear reactions among hydrogen isotopes takes place [1]. According to the most promising and practical nuclear fusion reaction, deuterium and tritium react to produce He and neutrons at temperatures of around 150 million °C and at low pressure in a toroidal plasma chamber:

D +T → ^4^He + n (1)

where n is a fast neutron (14.07 MeV).

Tritium is a radioactive isotope (half-life 12.32 y) that is produced in a Li-based breeding blanket surrounding the toroidal plasma chamber through two reactions:

n + ^7^Li → ^4^He + n′ (2)

n′ + ^6^Li → T + ^4^He (3)

where n′ is a thermal (slow) neutron.

**Figure 1:**
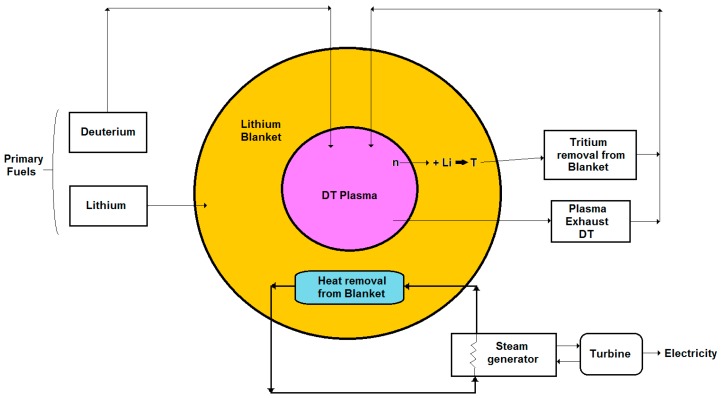
Schematic of a fusion power plant.

In the Li-blanket of a fusion power plant (see Figure 1), the neutron energy is converted into heat that is firstly transferred to the cooling system and then used to produce electricity, while the tritium produced has to be delivered to the plasma chamber in order to sustain the reaction (1). Accordingly, a fusion reactor needs only deuterium and Li as “external” fuels, which are both clean and widely available on the Earth. Deuterium can be obtained from water, where it is present with the isotopic ratio D/H of around 1/6000, while lithium can be taken from rocks and oceans [1].

Presently, an experimental tokamak designed to produce 500 MW fusion power is under construction in France in the framework of the international project ITER, which aimed to study the hydrogen isotopes’ plasmas and to test the main technologies [2]. In parallel, there are several on-going projects dedicated to the design and building of fusion facilities [3,4]. In particular, the EUROfusion Consortium is developing the project known as DEMO, which aims at realizing a reactor of fusion power 1500 MW (500 MWe) capable of demonstrating the production of electricity by operating with a “closed” fuel cycle [5].

Particularly, the fuel cycle of a fusion reactor consists of all the operations dedicated to the extraction and purification of the tritium from the breeding blanket, as well as the treatment of the gaseous and liquid streams containing the hydrogen isotopes [6]. With the realization of a “closed” fuel cycle, the tritium is confined to the fusion power plant in such a way that it can fulfill the requirement of safe production of clean energy.

This paper briefly reviews the fusion fuel cycle by focusing on the separation processes based on Pd membranes. Furthermore, the description of an innovative process that uses a Membrane Gas–Liquid Contactor (MGLC) for the extraction of tritium from liquid LiPb is described.

## 2. Fusion Fuel Cycle

As a rough estimation, a fusion reactor of 1 MWe needs to burn about 56 kg/year of tritium. The burning efficiency is around 1% (e.g., 99% of the DT mixture fed into the plasma chamber does not react and has to be treated by the fuel cycle before being re-sent to the plasma chamber); therefore, the total throughput of tritium is very large—about 5600 kg/year [7].

The feasible operation and safe management of a fusion reactor depends on the self-sufficiency of tritium and the control of the tritium releases [8,9,10]. Tritium self-sufficiency can be achieved by providing the T necessary to sustain the thermonuclear reaction, compensating the T inventory and losses in the fuel cycle systems and the 5%/year of T decay, thus providing a reserve for the start-up of new reactors. These requirements can be obtained by a fuel cycle characterized by very efficient processes for treating the plasma exhausts, extracting the tritium from the blanket and recovering the tritium from the coolant and other confinement systems. In this vein, the fuel cycle processes have to exhibit high availability and reliability and produce low quantities of wastes. 

**Figure 2:**
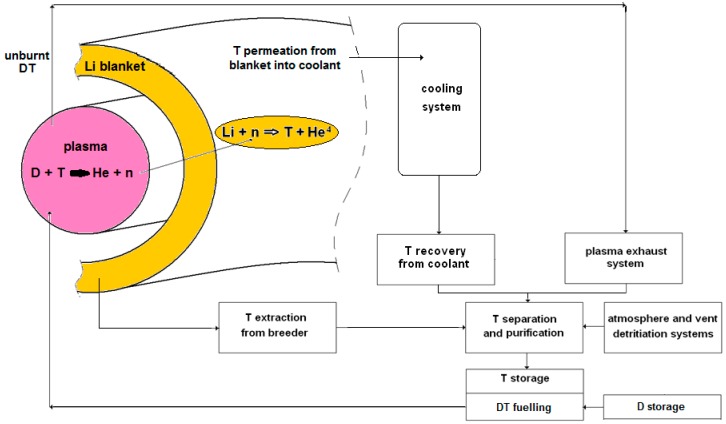
Schematic of the main subsystems of the fusion fuel cycle.

The schematic of Figure 2 shows the main subsystems of the fusion fuel cycle:

- storage of D and T,
- fueling of the DT mixture into the plasma,
- vacuum-pumping from torus and plasma exhaust treatment,
- tritium extraction from breeder (liquid LiPb or solid Li-ceramics),
- tritium extraction from coolant (namely water or He), and
- atmosphere and vent detritiation.

In these subsystems, tritium and other hydrogen isotopes are separated through three main categories of processes. First, the separation from the water is used as a coolant of the breeding blanket (where tritium permeates through the metal walls of the heat exchange systems) or from the water obtained by the oxidation of tritiated gases and other detritiation treatments (e.g., the atmosphere and vent detritiation systems). Then, the separation of tritium from helium is used as coolant in the breeding blanket, and the helium is used for purging the solid breeders (Li ceramics). Finally, the extraction of tritium from the liquid LiPb in the fusion reactor concepts where this material is used as a breeder.

### 2.1. Tritium Extraction from Water

Due to the slightly different boiling points of the hydrogen isotopes and their oxides, separation of tritium via water distillation exhibits low separation factors and needs large units with high energy consumption, which can be considered inconvenient [11]. 

Other processes are based on the isotopic exchange reactions, which take place among the hydrogen isotopologues in the molecular (H_2_, D_2_, T_2_, HD, HT, DT) and oxidized form (H_2_O, D_2_O, T_2_O, HDO, HTO, DTO). It is noteworthy that, in these exchange reactions, the thermodynamic equilibrium shifts towards the oxidized form. As an example, Table 1 reports the equilibrium constant of the reaction [12]:

HT + H_2_O ⇔ H_2_ + HTO (4)

Relying on this property, both the vapor phase catalytic exchange (VPCE) and liquid phase catalytic exchange (LPCE) processes use packed columns, where tritiated water exchanges with hydrogen. The separation factors D/T are 1.22 and 1.67 for VPCE (473 K) and LPCE (298 K), respectively, while analogously, the separation factors H/T are 2.13 (VPCE) and 7.14 (LPCE) [11].

Electrolysis of tritiated water can achieve higher separation factors (H/T = 10 at 353 K), and its application is proposed for treating tritiated water of high activity; however, it is characterized by high operational costs, and also presents safety concerns (risk of recombination of oxygen with tritiated gas). Accordingly, in the Combined Electrolysis and Liquid Phase Catalytic Exchange (CECE), the bottom stream of a VPCE column, enriched in tritium, is sent to an electrolyzer, whereby the load of the electrolysis is reduced and high decontamination factors can be achieved [13]. The use of Pd membranes for tritium extraction from water is described below (Section 3).

### 2.2. Tritium Extraction from Helium

The tritium and the other hydrogen isotopes contained in helium are used as purging gas for the solid breeders (Li-ceramics) or as a coolant in the blankets. Their removal from He can be realized by selective absorption (at room or cryogenic temperature) onto molecular sieve beds [14]. These systems can treat high flowrates but work in batch mode, thus increasing the tritium inventory of the plant. Alternatively, when the tritium concentration in helium is low, it can be convenient to oxidize the stream, and then separate (e.g., by cold traps) the tritiated water that is then treated as described previously (Section 2.1).

### 2.3. Tritium Extraction from LiPb

When produced by the reactions (2) and (3) into a liquid breeder, the tritium is solubilized into the eutectic alloy, LiPb. Extraction from the liquid metal is performed via gas–liquid contactors consisting of vertical columns where a helium stream rises up counter-currently to the LiPb fed at the top (300–500 °C). The gas–liquid contactors can be realized by packed bed columns, bubble columns, or spray columns [15,16]. Thanks to their high packing area (750 m^2^ m^−3^), the packed column can achieve 30% efficiency (the ratio between the exchanged hydrogen flux and the hydrogen flux in the inlet LiPb) [17].

**Figure 3:**
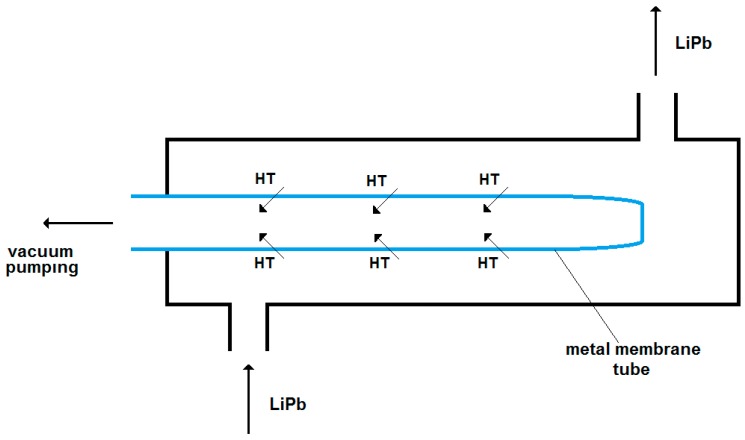
Schematic of the Permeation Against Vacuum (PAV) concept.

Recently, the use of metal membranes has been introduced through the development of the Permeation Against Vacuum (PAV) concept, depicted schematically in Figure 3. In preliminary studies [18,19], the LiPb was shown to flow inside a cylindrical and concentric multi-channel metal membrane, where from the liquid LiPb, tritium which permeated through the metal wall was collected in the permeate side by vacuum pumping. Materials such as α-Fe and refractory metals (V, Nb, Ta) are under consideration for this kind of membrane that has to work at a high temperature in contact with liquid metal flow. For instance, with V membranes, extraction efficiency values of up to 39% have been assessed [20]. Further testing of PAV will be needed to verify the tritium extraction capability as well as the materials’ stability, which could be affected by the erosion-corrosion of LiPb at high temperatures.

## 3. Pd Membranes in the Fusion Fuel Cycle

With respect to traditional processes, the membrane systems are characterized by high reliability and continuous operations and, therefore, their application in the fuel cycle can ensure the efficient and safe management of a fusion power plant [21].

Pd membranes have been proposed for tritium recovery from plasma exhausts, where a membrane reactor concept, the PERMCAT, has been studied [22,23]. In this Pd-membrane reactor, plasma exhausts, consisting of tritiated gases (HTO, CH_3_T, etc.) mixed with the unburnt DT are sent into the lumen (or shell) of a Pd-Ag tube, where, over a catalyst, the isotopic exchange reactions take place with a protium (H_2_) stream, flowing counter-currently in the side of the shell (or lumen). Examples of such reactions are:

CH_3_T +H_2_ ⇔ CH_4_ + HT (5)

H_2_ + HTO ⇔ H_2_O + HT (6)

Due to the complete selectivity of the Pd-Ag tube used, only the hydrogen isotopes can permeate through the membrane and, according to the reaction and permeation kinetics defined by the materials (e.g., catalyst, membrane) and the geometry of the reactor (membrane area, tube wall thickness), the isotopic exchange among the hydrogen isotopes allows for high decontamination of the tritiated feed stream to be attained [24,25].

**Figure 4:**
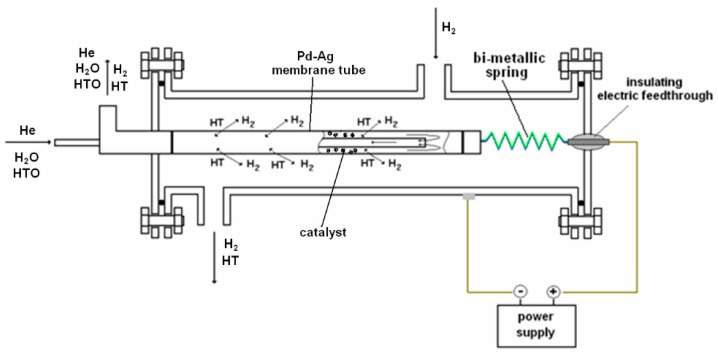
Scheme of the membrane reactor powered by direct ohmic heating (“Reprinted from Fusion Engineering and Design vol. 86, S. Tosti, C. Rizzello, F. Borgognoni, N. Ghirelli, A. Santucci, P. Trabuc, Design of Pd-based membrane reactor for gas detritiation, Pages 2180–2183, Copyright (2011), with permission from Elsevier.”) [26].

Recently, a Pd-membrane reactor has been developed for recovering tritium from tritiated water coming from the detritiation of laboratory wastes [26,27]. A Pd-Ag tube (with a length of 500 mm, wall thickness of 0.150 mm, and diameter of 10 mm) has been assembled in a finger-like configuration into a stainless-steel shell. The direct ohmic heating of the membrane tube allowed quick temperature ramps to be achieved, even with modest electrical power. As shown in Figure 4, this device works like a PERMCAT-type reactor, where the exchange among the hydrogen isotopes occurs over the catalyst, and the tritium permeated through the selective membrane is then collected in the side of the shell.

This membrane reactor has been simulated by a finite element code under the hypotheses of perfect gas behavior, negligible axial and radial diffusion (plug flow fluid dynamic regime), negligible pressure losses, and reaction (6) at equilibrium conditions. The code runs several iterations, starting with a tentative partial-pressures profile in both the lumen (reaction) and shell (permeate) side. Then, the permeation fluxes of H_2_ and HT were evaluated in each finite element through the formulas [28]:

(7) $$J_{H_{2}}=\frac{{\mathrm{Pe}}_{H_{2}}}{\mathrm{th}}\left(x_{H_{2\;\mathrm{up}}}\;\sqrt{p_{H_{2\;\mathrm{up}}}+p_{{\mathrm{HT}}_{\mathrm{up}}}\;}-x_{H_{2\;\mathrm{down}}}\;\sqrt{p_{H_{2\;\mathrm{down}}}+p_{{\mathrm{HT}}_{\mathrm{down}}}}\right)$$

(8) $$J_{\mathrm{HT}}=\frac{{\mathrm{Pe}}_{\mathrm{HT}}}{\mathrm{th}}\left(x_{{\mathrm{HT}}_{\mathrm{up}}}\;\sqrt{p_{H_{2\;\mathrm{up}}}+p_{{\mathrm{HT}}_{\mathrm{up}}}\;}-x_{{\mathrm{HT}}_{\mathrm{down}}}\;\sqrt{p_{H_{2\;\mathrm{down}}}+p_{{\mathrm{HT}}_{\mathrm{down}}}}\right)$$

where: J is the permeation flux (mol m^−2^ s^−1^) through the membrane area of the finite element, Pe the permeability coefficient (mol m^−1^ s^−1^ Pa^0.5^), th the membrane thickness (m), and p (Pa) is the partial pressure. The indexes H_2_ and HT are referred to as the hydrogen isotopic species and as the indexes up and down to the upstream (lumen) and downstream (permeate) side.

Once the permeation flux of the hydrogen isotopes has been assessed, the mass balance over a finite element “i” in the feed side for the species “m” can be carried out by the expression:

(9) $$N_{m,\;i}=N_{m,\;i-1}+r_{m,i}\;V-{k\;J}_{H,i}\;A$$

where N is the molar flow rate (mol s^−1^), r are the moles generated (mol m^−3^ s^−1^) in the finite element according to the reaction (6), J_H,i_ the permeation flux of the hydrogen isotopes (H_2_ or HT), k is a coefficient equal to 1 for the hydrogen isotopes (H_2_ and HT) and equal to zero for the other species, and V is the volume of the finite element (m^3^) and A its permeation area (m^2^).

The mass balance in the permeate side considers the two cases of co-current and counter-current mode:

(10) $$N_{H,\;i}=N_{H,\;i\pm 1}+J_{H,i}\;A$$

where $N_{H,\;i+1}$ and $N_{H,\;i-1}$ are used for the counter-current and co-current case, respectively.

The profiles of the hydrogen isotope (H_2_ and HT) flow rates obtained in the current iteration can be compared with those calculated in the previous one until convergence is reached.

From the results of the simulation code, the decontamination factor (DF) of the membrane reactor can be evaluated as:

(11) $$\mathrm{DF}=\frac{{\mathrm{HTO}}_{\mathrm{in}}}{{\mathrm{HTO}}_{\mathrm{out}}+{\mathrm{HT}}_{\mathrm{out}}}$$

where HTO_in_ are the moles of tritiated water feeding the membrane reactor, and HTO_out_ and HT_out_ are the moles of tritiated water and the moles of HT_out_ leaving the membrane reactor with the retentate stream.

When feeding the shell side with 100 NmL/min of protium at 400 °C, the code assessed a DF of 9.23 for the Pd-membrane reactor operating counter-currently [26]. Under similar operating conditions (feed and shell pressure of 130,000 kPa and 25 mbar, respectively), in experimental tests, this reactor exhibited around 10 decontamination factors [29].

Pd-membrane reactors for separating tritium from tritiated water both via isotopic exchange and the water–gas shift reaction have been studied [30,31]. Detritiation of water via isotopic exchange relies on the reaction (4), while the water–gas shift reaction of HTO can be described by:

CO + HTO ⇔ CO_2_ + HT (12)

From the model analysis, for Pd tubes with a wall thickness of 0.100 mm, the decontamination factors attained by the water–gas shift reaction are at least one order of magnitude higher than those achieved by the isotopic exchange [30]. As a concern, side-reactions could make the water–gas shift process unfeasible for fusion application. In particular, the formation of tritiated methane by CO and CO_2_ methanation has to be avoided:

CO + 3Q_2_ ↔ CQ_4_ + Q_2_O (13)

CO_2_ + 4Q_2_ ↔ CQ_4_ + 2Q_2_O (14)

where Q can mean H or T.

For this reason, specific catalysts for the water–gas shift reaction have been studied and tested in experiments carried out with heavy water (HDO), where a specific Pt-based catalyst, developed by the Instituto de Investigaciones en Catálisis y Petroquímica (Santa Fe, Argentina) [32], exhibited low formation of methane (at max. 0.35% at 200 kPa and 400 °C) with the capability to recover more than 80% of the deuterium which was fed [33].

## 4. Porous Membranes for Tritium Extraction from LiPb

The tritium generated in the LiPb breeding blanket by the nuclear reactions (2) and (3) was solubilized in the liquid metal. Sieverts’ law establishes the relationship between the concentration of the hydrogen isotopes in the metal with the partial pressure of hydrogen isotopes in the gas phase under equilibrium conditions:

C_H_ = K_S_ P^0.5^ (15)

where C_H_ is the concentration of the hydrogen isotopes in the LiPb (at. fraction m^−3^), K_S_ is the Sieverts’ constant (at. fraction m^−3^ Pa^−0.5^), and P is the hydrogen isotopes’ partial pressure in the gas phase (Pa). Typical values of K_S_ for hydrogen into LiPb are 10^−8^–10^−6^ at. fraction Pa^−0.5^ in the temperature range 300–1000 °C [34].

As discussed previously, PAV-type permeators have been studied for the extraction of tritium solubilized in the LiPb. PAV devices offer advantages typical of membrane processes (continuous operation, high extraction efficiency, simplified operation); however, these permeators have to face problems related to the manufacturing of complex metal structures. Furthermore, the membrane materials have to exhibit high hydrogen permeability, as well as resistance to the corrosion under the operating conditions (high temperature, contact with flowing LiPb) [18].

**Figure 5:**
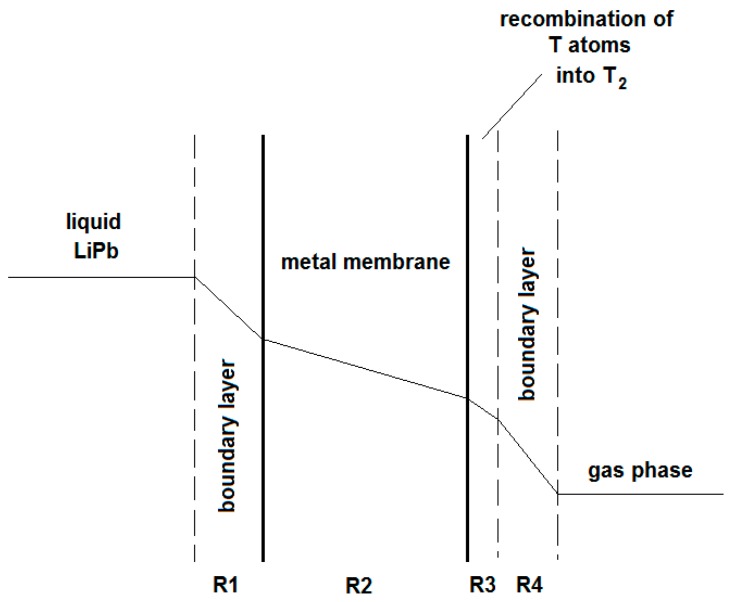
PAV permeator: series of mass-transfer resistance in the extraction of hydrogen isotopes, from liquid LiPb to the gas phase.

The hydrogen mass transfer from the LiPb to the gas-phase downstream metal membrane is schematically depicted in Figure 5. The main mass-transfer resistances are due to the tritium diffusion through the boundary layer of the liquid metal (R1), the diffusion through the metal wall (R2), the recombination of T atoms into molecules over the downstream metal membrane’s surface (R3), and the tritium diffusion through the boundary layer of the gas phase (R4). In particular, under the hypothesis of vacuum-pumping downstream the PAV-permeate side, the diffusion through the metal wall is governed by Fick’s law:

J = Pe_M_ P^0.5^ th^−1^ (16)

where J (mol m^−2^ s^−1^) is the hydrogen permeation flux, Pe_M_ is the permeability coefficient (mol m^−2^ s^−1^ Pa^−0.5^), and th is the metal membrane wall thickness (m).

Since the PAV has to withstand the erosion-corrosion of the liquid metal, the Pd-Ag alloys cannot be used for the membrane. The alternative metal membranes have showed the following drawbacks:

- α-Fe has low hydrogen permeability (7.99 × 10^−11^ mol m^−2^ s^−1^ Pa^−0.5^ at 400 °C) [35];
- refractory metals (i.e., Nb and V) exhibit hydrogen permeability higher than Pd-alloys (e.g., 3.59 × 10^−6^ and 3.40 × 10^−7^ mol m^−2^ s^−1^ Pa^−0.5^ at 400 °C for the Nb and V, respectively) [35], but are characterized by high hydrogen solubility, thus involving low durability (embrittlement) and safety concerns (high tritium inventory).

### Membrane Gas–Liquid Contactors

In a new approach [36], porous membranes to extract tritium from LiPb were studied. The liquid LiPb behaves as a non-wetting (or partial wetting) liquid, meaning it can partially penetrate the pores of the membrane without entering into the gas phase, where leaks of liquid metal cannot be accepted.

**Figure 6:**
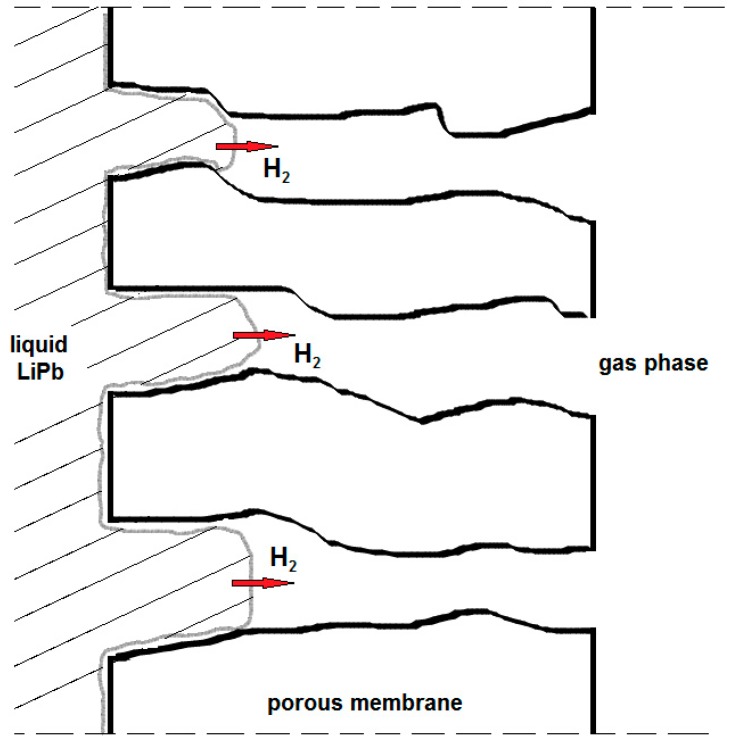
In the Membrane Gas–Liquid Contactor (MGLC) realized by porous membranes, the liquid LiPb partially penetrates the pores of the membrane without entering into the gas phase.

This system can be defined a Membrane Gas–Liquid Contactor (MGLC), where the interface between the liquid LiPb and the gas phase is realized inside the membrane pores (see Figure 6). Compared to the PAV concept, the MGLC exhibits mass-transfer resistance through the porous membrane, instead of through the metal membrane (R2 of Figure 5).

In order to design a porous membrane acting as a gas–liquid contactor for the extraction of tritium from the liquid LiPb, the size of the (cylindrical) pores was assessed through the Washburn equation used in porosimetry. This expression establishes the equilibrium between the external pressure forcing the liquid into the pores and the resistance given by the liquid’s tension surface:

(17) $$P_{L}-{\;P}_{G}=-\;\frac{4\;\gamma \cos\theta \;}{D}$$

where P_L_ and P_G_ are the pressure of the liquid and the gas phase (Pa), γ is the surface tension of the liquid metal (N m^−1^), θ is the contact angle of the intrusion liquid over the porous material (usually, a value of 140° can be assumed for liquid metals over most solids), and D is the pore diameter (m).

The surface tension of the LiPb in the temperature range of 247–727 °C is expressed by [37]:

(18) $$\gamma \;=0.52-0.11\times \;10^{-3}\;T$$

with γ in N m^−1^ and T, temperature, in K.

## 5. Results and Discussion

A first-time application of the Membrane Gas–Liquid Contactor was shown to be related to the extraction of tritium from LiPb blankets as opposed to the PAV devices. By applying the Formulas (17) and (18), it resulted that a membrane with a pore size of about 3–7 μm was non-wetted or partially wetted when used for the extraction of tritium from liquid LiPb at 300–500 °C and 200–400 kPa.

**Figure 7:**
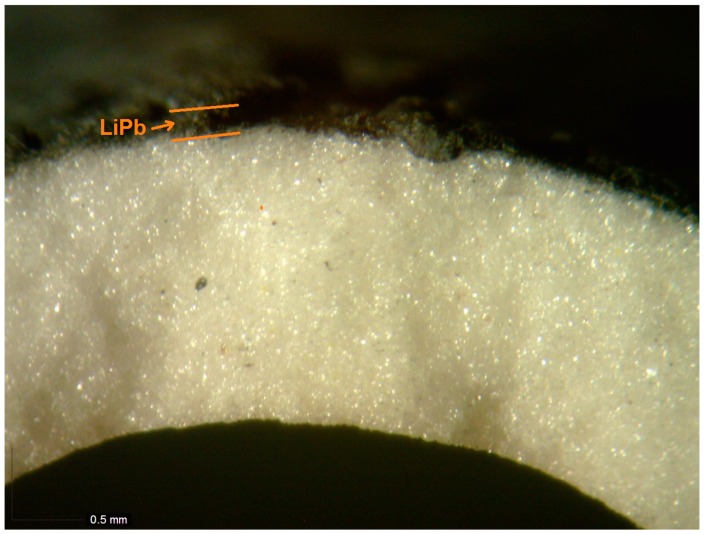
View (100×) of the cross-section of a porous ceramic membrane after immersion in LiPb at 450 °C.

Under testing, a ceramic porous tube made of alumina of 40 mm in length, ext./int. diameters of 7/10 mm, and a pore size of 10 μm was immersed in liquid LiPb at 450 °C. As shown in Figure 7, the LiPb partially penetrated the pores without passing the membrane wall. In this case, the difference of the pressures $\left(P_{L}-{\;P}_{G}\right)$ is given by the hydrostatic pressure of the height of the LiPb that corresponds to the few tens of kPa. In fact, under these conditions, the pore size calculated by the Formula (17) is shown to be around 10 μm.

As discussed, the mass-transfer mechanism of the tritium through such a kind of membrane can be obtained from the PAV one by changing the resistance R2 (diffusion through the metal lattice) with the mass-transfer resistance through the pores of the MGLC (gas diffusion). The mass-transfer mechanism (Poiseuille or Knudsen) is established by comparing the free mean path (λ) of the tritium molecules and the pore size of the membrane. A preliminary assessment has been performed for a membrane of pore size 10 μm, operating at 400 °C and 200–400 kPa. Under these conditions, the free mean path of the tritium molecules is smaller than the pore size and, therefore, the mass transport is ruled by the Poiseuille regime, and the gas permeability Pe (mol m^−1^ s^−1^ Pa^−1^) can be assessed by the expression [38]:

(19) $$\mathrm{Pe}=\frac{{\;r}^{2}}{8\;\mu \;R\;T\;}{\;p}_{\mathrm{av}}$$

where:

ε is the porosity;
η is the shape factor;
r is the pore radius, m;
p_av_ is the mean pressure, Pa;
µ is the viscosity, Pa s;
R is the gas constant, 8.314 J K^−1^ mol^−1^; and
T is the temperature, K.

Table 2 reports the values of the free mean path and of the permeability calculated by Formula (19).

In principle, the thickness of the metal wall of the metal membranes in the PAV concept and the thickness of the MGLC are very similar (1–2 mm). Thus, the calculated gas permeability through the porous membrane leads to mass-transfer resistance lower than the values of R2 of the PAV, using Nb and V as membrane materials.

Furthermore, when compared to the PAV concept, ceramic MGLC can exhibit better resistance to erosion-corrosion of the LiPb flow at high temperatures, and is characterized by a lower hydrogen inventory.

### Sensors for Detecting Tritium in LiPb

Another application of MGLC in nuclear fusion technology concerns the development of sensors for measuring the tritium concentration into liquid LiPb. This measurement is mandatory for the correct operation of a tokamak and, in particular, for the control of the tritium inventory in the Li-breeding blanket. Based on the PAV concept, membrane tubes made of iron or Nb have been studied for the measurement of the tritium into LiPb [39,40]: these tubes are immersed into liquid LiPb and their lumen side is vacuum-pumped and connected to a detecting system (e.g., a mass spectrometer). Neglecting the mass-transfer resistance due to the boundary layers and the recombination reactions, from the expressions (15) and (16), it is possible to establish the relationship between the concentration of the tritium into the LiPb and the permeation flux measured by the detecting system:

C_H_ = K_S_ J th/Pe_M_ (20)

The tests have shown a quick degradation of the Nb sensor (the permeation flux decreased during testing due to the oxidation of the membrane surface) while the iron sensor could not be easily operated in steady-state conditions because of the long time required to achieve the hydrogen pressure equilibrium [39]. In this view, a porous ceramic membrane made of materials characterized by low reactivity at high temperatures (alumina, zirconia, SiC, etc.) could work effectively as a sensor for measuring the tritium concentration. As discussed for the application of the MGLC, the absence of the metal membrane should ensure low mass-transfer resistance and a quick response time of the sensors.

## 6. Conclusions

Exploitation of nuclear fusion via magnetic-confined devices (tokamak) relies on the development of an efficient and reliable fuel cycle. In future tokamaks, tritium will be produced in a Li-based breeding blanket; here, it has to be extracted and purified before being sent to the plasma chamber for sustaining the nuclear fusion reaction with deuterium.

This paper has reviewed the main operations dedicated to the extraction and purification of the tritium from the breeding blanket, as well as the treatment of the gaseous and liquid streams containing the hydrogen isotopes. Particular focus has been given to the membrane systems that are characterized by high reliability and continuous operations and, therefore, their application in the fuel cycle can ensure the efficient and safe management of a fusion power plant.

Processes using self-supported membranes made of Pd-alloys have been modelled and characterized for the recovery of tritium and the other hydrogen isotopes from both gaseous and liquid streams. Thanks to their complete (infinite) selectivity to hydrogen isotopes, this kind of membrane allows for a high level of separation factors to be achieved in highly efficient operations. Successful applications of Pd-membrane reactors have been reported in water detritiation through both water-gas shift and isotopic swamping reactions. A simplified model code has been developed for simulating a membrane reactor using a Pd-Ag self-supported tube, and the results, in terms of decontamination factors, have been verified by experiments.

Finally, an innovative Membrane gas Liquid Contactor (MGLC) has been studied for the extraction of tritium from the liquid LiPb, proposed as a candidate blanket material for future tokamaks. This device uses porous ceramic membranes that are partially wetted or non-wetted by the liquid metal, and in such a way, the hydrogen isotopes’ mass transfer takes place through the interface between the liquid LiPb and the gas phase inside the membrane pores. Compared to the alternative PAV concept that uses dense metal membranes, MGLC, made of ceramic membranes, may reveal better resistance to the erosion corrosion of the LiPb flow at high temperatures. Furthermore, the same MGLC can be applied as a sensor for detecting the concentration of the hydrogen isotopes into the LiPb by exhibiting low mass-transfer resistance and a quick response time.

## Figures and Tables

**Table 1:** Equilibrium constant of reaction (4), from [12].

T, °C	Equation Constant
16.0	6.47
20.2	6.24
25.0	6.01
56.2	4.84
79.6	4.23
111.2	3.64
158.4	3.03
217.1	2.54
302.9	2.08

**Table 2:** Calculated tritium permeability through the pores of the MGLC at 400 °C.

Pressure, kPa	λ, μm	Gas Permeability, mol m^−1^ s^−1^ Pa^−1^
200	0.124	1.65 × 10^−5^
400	0.062	3.26 × 10^−5^
